# The Role of Liquid-Liquid Phase Separation in the Accumulation of Pathological Proteins: New Perspectives on the Mechanism of Neurodegenerative Diseases

**DOI:** 10.14336/AD.2024.0209

**Published:** 2024-04-20

**Authors:** Xingyu Lu, Jiongtong Lu, Shengnan Li, Sifan Feng, Yan Wang, Lili Cui

**Affiliations:** ^1^Guangdong Key Laboratory of Age-Related Cardiac and Cerebral Diseases, Affiliated Hospital of Guangdong Medical University, Zhanjiang, China.; ^2^The Marine Biomedical Research Institute of Guangdong, School of Ocean and Tropical Medicine, Guangdong Medical University, Zhanjiang, Guangdong, China.

**Keywords:** liquid-liquid phase separation, neurodegenerative diseases, Tau, TDP-43, FUS, α-Syn, HTT

## Abstract

It is widely accepted that living organisms form highly dynamic membrane-less organelles (MLOS) with various functions through phase separation, and the indispensable role that phase separation plays in the mechanisms of normal physiological functions and pathogenesis is gradually becoming clearer. Pathological aggregates, regarded as hallmarks of neurodegenerative diseases, have been revealed to be closely related to aberrant phase separation. Specific proteins are assembled into condensates and transform into insoluble inclusions through aberrant phase separation, contributing to the development of diseases. In this review, we present an overview of the progress of phase separation research, involving its biological mechanisms and the status of research in neurodegenerative diseases, focusing on five main disease-specific proteins, tau, TDP-43, FUS, α-Syn and HTT, and how exactly these proteins reside within dynamic liquid-like compartments and thus turn into solid deposits. Further studies will yield new perspectives for understanding the aggregation mechanisms and potential therapeutic strategies, and future research directions are anticipated.

## 1. Introduction

Neurodegenerative diseases include a range of diseases characterized by progressive degeneration and death of neurons, resulting in damage to the central nervous system and cognitive and motor impairment. These diseases manifest in different clinical symptoms with unknown aetiology and are publicly perceived as being difficult to cure. Interestingly, insoluble inclusions are widely observed in these diseases and inferred as the main culprit in neurodegeneration pathology, such as microtubule-associated protein Tau (MAPT) cytosolic aggregates in AD (Alzheimer’s disease), alpha-synuclein (α-Syn) inclusion bodies or Lewy bodies in PD (Parkinson disease), TARDNA-binding protein 43 (TDP-43) or FUS intracytoplasmic inclusion bodies in ALS (amyotrophic lateral sclerosis) and FTD (frontotemporal dementia), and polyglutamine (PolyQ) aggregates in HD (Huntington disease). Pathological protein aggregates contain disease-specific proteins that the organism recognizes as misfolded proteins. The process of aggregation cannot be performed in one step, and unfolded proteins transform to multiple conformational states by forming substable, transient prefibrillar intermediate species. Growing evidence indicates that this conformational transformation involves a process known as phase separation, in which proteins are locally concentrated into dynamic liquid-like compartments and contribute to the transformation of soluble proteins to insoluble amyloid fibrils. The concept of condensates has recently emerged, providing us with a novel perspective on neurodegenerative diseases [1-3].

In this review, we focus on the significant role that phase separation plays in cell biology, particularly its implications for neurodegenerative diseases, and how it impacts disease pathology. We anticipate that a deeper understanding of the mechanism of phase separation will make a difference in the stagnant progress of disease treatment.

## 2. Liquid-liquid Phase Separation (LLPS)

**Figure 1.**
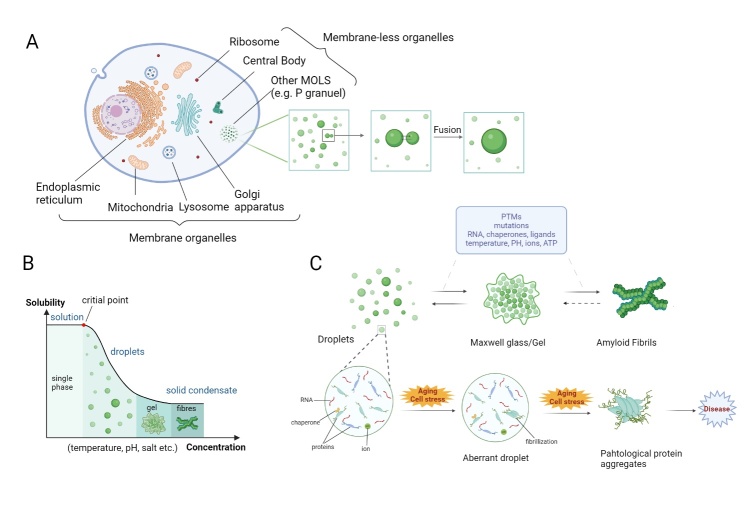
**Liquid-liquid phase separation**. (**A**) Liquid-liquid phase separation in cells. In addition to membrane organelles, living cells contain many membraneless organelles (MLOS) assembled by phase separation [11]. Under certain conditions, biomolecules such as proteins and RNA condense to form small droplets, which fuse into larger droplets and gradually assemble into MLOS. (**B**) Two phases coexist in one system when the concentration increases, and droplets will transition into gel or amyloid fibrils. (**C**) Modulated by several factors, LLPS droplets can form gels or irreversible amyloids. Under pathological conditions, aberrant phase separation results in the formation of undesirable droplets and the subsequent aggregation of proteins.

The cell is the structural and functional unit where a complex mass of biochemical components, such as proteins, nucleic acids, and lipids, are dispersed. Membrane-bound organelles (such as the endoplasmic reticulum, Golgi apparatus, and mitochondria) enable these molecules to locate their exact companions and interact in a disciplined and autonomous fashion in a specific space. In addition, researchers have recognized numerous membraneless "compartments" in cells, such as the nucleolus [4, 5], P granules [6], and stress granules [7, 8]. These membraneless organelles, also known as "biomolecular condensates", have liquid-like properties, allowing them to deform, reorganize and fuse freely, facilitating the aggregation and exchange of materials with the external environment, which is closely related to the concept of liquid-liquid phase separation (LLPS) [9-11]. LLPS is the self-assembly of various biomolecular droplets in a consistent system (such as cytoplasm or nucleoplasm), creating two separated liquid phases with a high concentration of molecules in one phase and a relatively dilute concentration in another (Fig. 1).

### 2.1 Phase separation plays an essential role in physiological functions

LLPS, a common cellular mechanism, plays a fundamental role in a variety of biological functions. Recently, more evidence has been put forwards to emphasize the prevalence and importance of phase separation in the activities of living organisms, including chromatin aggregation, cellular signal transduction[12], and DNA repair[13]. Keizer reported that chromosomes were almost liquid-like during interphase in cell division undergoing LLPS [14]. Zheng confirmed the indispensable role of phase separation in regulating centrosome maturation, spindle assembly and polarity establishment during cell division [15]. The observation of biomolecular condensates at different stages of the transcription cycle emphasized the pivotal role that LLPS plays in gene transcription, facilitating the dynamic assembly of transcription regulators [16]. The human adenovirus (Adv) 52-kDa protein has been revealed to assemble through phase separation, which plays a key role in the process of viral infection [17].

*The assembly of condensates.* Mostly proteins and RNA are involved in phase separation, and current research is focused mainly on proteins. Biomolecular condensates are not just aggregations of single proteins; they may have complex compositions with hundreds of components, known as complex coalescence [18]. For instance, the P particle components PGL-1, PGL-3, and LAF-1 can phase separate individually [19]. Notably, not all the components appear to play a pivotal role in the assembly of the condensates. Some resident biomolecules with multiple interacting motifs that can undergo LLPS are commonly referred to as ‘scaffolds’, which are vital for the structural integrity of condensates. The other components recruited into the condensate driven by LLPS are considered 'clients' [20], which are dispensable for condensate assembly and are dictated by scaffolds to some extent.

*Driving force of phase separation.* It has been reported that the liquid-liquid phase separation of biomolecules is generally driven by multiple weak interactions of different types, including electrostatic interactions, hydrophobic interactions, cation-pi interactions and pi-pi stacking [21]. One of the most common interactions is driven by the low-complexity domain (LCD), usually known as the intrinsically disordered region (IDR), which refers to domains/regions containing a small set of repeating amino acids [19]. Intrinsically disordered proteins (IDPs) do not have a globular structure and are vulnerable to environmental changes. They can perform a wide range of biological functions, including mediating LLPS [22, 23]. The presence of an intrinsically disordered region (IDR) in a protein is commonly used to evaluate or even determine its potential for phase separation. These disordered regions are enriched with aromatic amino acids and charged amino acids such as arginine, which can generate stronger multivalent forces and drive LLPS in response to changes in the function or concentration of IDPs [1]. Additionally, it has been proposed that proteins containing multiple linear modules can induce phase separation through interactions between these functionally similar modules [24], and Li indicated that proteins with tandem repeats of a single Src homology 3 (SH3) domain and proteins composed of proline-rich motif (PRM) ligands can undergo phase separation in vitro [25]. In addition, the oligomerization domains of proteins may play a constructive role in driving the formation of condensates by increasing multivalent interactions [26-29]. The oligomeric domain of certain proteins, such as PopZ in bacteria, can form condensates whose solubility is modulated by other domains (IDRs) [30, 31].

*Initiating and regulating factors.* The initiation and regulation of LLPS involves a variety of factors, such as temperature [32], pH, salt ions, and ATP [33, 34]. When distributed in the intracellular environment at a high concentration, biomolecules such as proteins and nucleic acids will condense to reach a critical value due to multivalent intermolecular interactions and volume-exclusion effects when reaching the minimum concentration for phase separation [35, 36]. It has also been reported that posttranslational modifications (PTMs), such as phosphorylation and/or methylation, alter the saturation concentration of proteins by changing multivalent interactions [37, 38], and protein quality control systems (QCSs) are increasingly considered to play an important role in the assembly, disassembly, and maintenance of the dynamic equilibrium of condensates [39, 40]. The precise regulation and accurate proportion of all these factors work together and bring about the marvellous phenomenon of LLPS under certain conditions.

### 2.2 Aberrant LLPS may be involved in disease pathology

Abnormal aggregates disrupt the stability of proteins. Under external stress or genetic mutations, aberrant phase separation results in the formation of undesirable droplets. These “ageing” condensates change from the initial highly fluid liquid state to a more viscoelastic and rigid state, such as a gel or glass, and inevitably turn out to be solid amyloid fibrils with cytotoxicity, also known as amyloid plaques, in neurodegenerative diseases. Phase separation is involved in the activation and inhibition of oncogenes, and dysregulation of condensates has been observed in a variety of cancers. Point mutations, deletions, insertions, and fusions are often observed in the genomes of cancer cells, and phase separation is involved in genomic stability [41-43]. In addition, phase separation is involved in regulating the onset and progression of infectious diseases. Viral proteins involved in replication can phase separate to drive the formation of viral compartments [44]. α-Crystallin can form condensates through LLPS upon stimulation by several risk factors, such as ageing and diabetes, contributing to cataracts [45].

**Table 1:** Functions and pathogenic mechanisms of neurodegenerative disease-related proteins.

	Disease	Location	Mutations	Pathological manifestations	PTMs	Functions	Reference
**Tau**	Alzheimer's disease (AD)	Temporoparietal, hippocampus	MAPT (p301L, P301S, ΔK280, A152T)	Aβ plaques neurofibrillary tangles	Phosphorylation Acetylation Ubiquitination	bind to microtubules, contribute to microtubule condensation and assembly	[172] [56] [68] [69]
**TARDNA-binding protein-43 (TDP-43)**	Amyotrophic lateral sclerosis (ALS) Frontotemporal dementia (FTD)	Motor cortex Frontotemporal	TARDBP (A315T, Q331K, M337V, D169G, K181E, K263E)	stress granule	Phosphorylation Ubiquitination	mediate essential RNA processing	[96] [173] [174] [175] [176] [27]
**FUS**	ALS FTD	Motor cortex Frontotemporal	FUS (G156E, R244C)	stress granule	Phosphorylation Ubiquitination	mediate essential RNA processing	[7] [177] [98]
**alpha-synuclein (α-Syn)**	Parkinson's disease (PD)	Midbrain	SNCA (A53T, A53E, A30P, E46K, A18T, A29S)	Lewy bodies	Phosphorylation Ubiquitination	bind to the membrane and play a role in vesicle transport	[178] [179] [180] [181] [182] [183]
**Huntington (HTT)**	Huntington's disease (HD)	Basal ganglia	HTT (P703L, F2717L)	Neural loss astrocytosis	Phosphorylation Acetylation Methylation	may play a role in microtubule-mediated transport or vesicle function	[184] [185] [49]

## 3. LLPS of critical pathogenic proteins in neurodegenerative diseases

**Figure 2.**
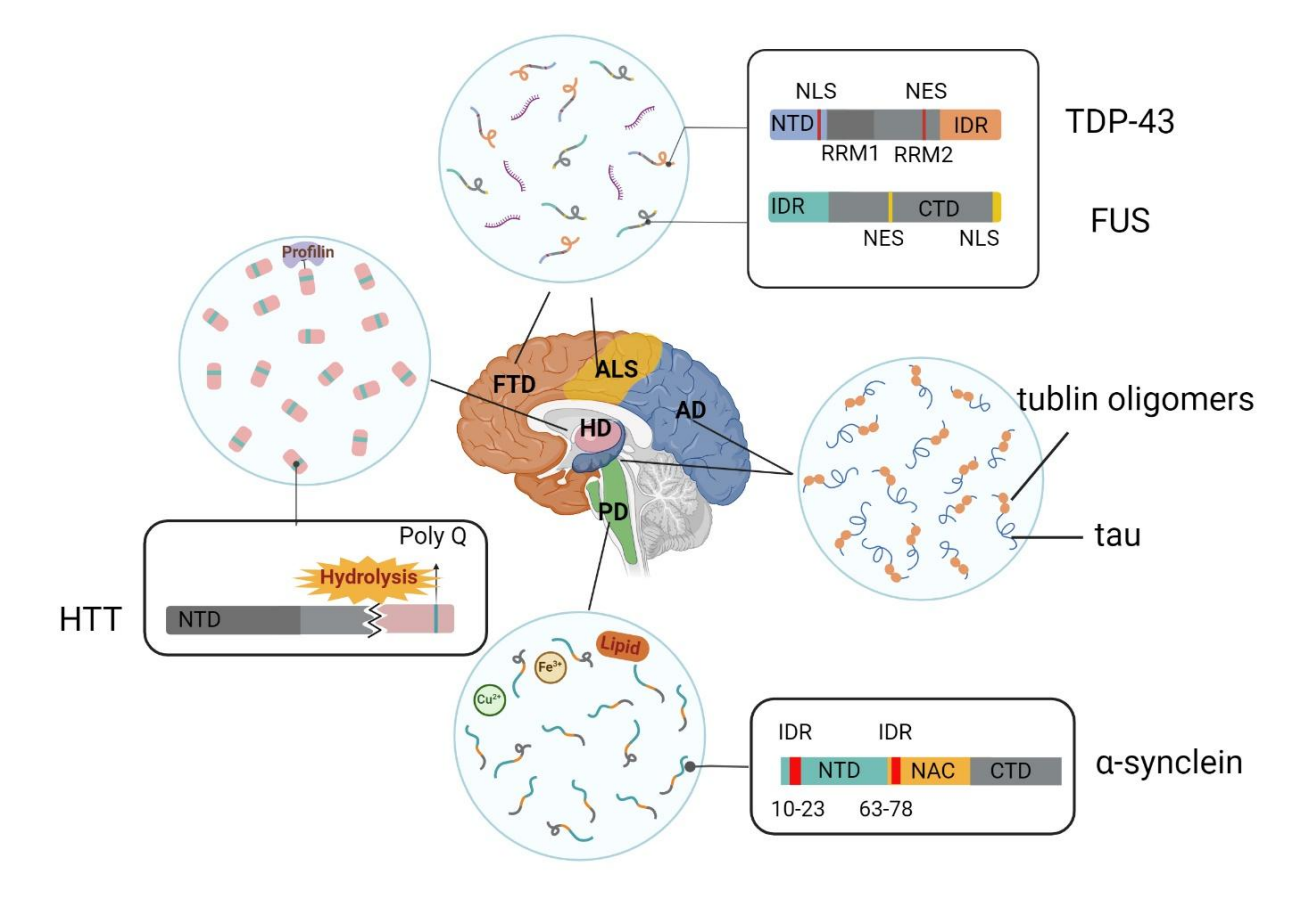
**LLPS in neurodegenerative diseases**. Under pathological conditions, disease-associated proteins undergo aberrant LLPS to form pathological aggregates, which may vary depending on the proteins involved and the disease context. The phase behaviour of proteins can be modulated by metal ions, molecular chaperones, and RNA through alterations in their charge or conformation. TDP-43 contains a nuclear localization signal (NLS) within its N-terminal domain (NTD) and a nonfunctional nuclear export signal (NES) in its RNA recognition motif (RMR) [47]. FUS contains an NES within its RRM as well as an NLS within its C-terminal domain (CTD) [48]. Proteolysis of the mutant Huntington protein results in the production of an exon 1 fragment, which accumulates in the nucleus of Huntington's disease neurons [49]. The NAC of α-Syn drives aggregation, while the IDR is located mainly in the NTD [50].

Gene mutations may contribute to the development of certain hereditary neurodegenerative disorders, but the pathogenesis of most sporadic neurodegenerative diseases, which account for the majority of these disorders, is poorly understood. Ageing and cellular stress can potentially induce alterations in protein conformation or stability, and as hallmarks of neurodegeneration, protein aggregates such as tau, TDP-43, FUS, α-Syn, and Htt are deposited in specific brain regions in a misfolded conformation, leading to progressive damage and dysfunction of neuronal cells (Table 1). Recent studies have revealed that these proteins are assembled into condensates through aberrant phase separation and transform into insoluble pathological inclusions with time, promoting the development of neurodegenerative diseases. Under physiological conditions, these proteins exhibit dynamic interconversion between dispersed phases, droplets, and reversible gels. Although neurodegenerative diseases exhibit diverse clinical manifestations and aetiologies, the presence of pathological inclusions provides promising avenues. Pathologic protein deposits may vary depending on the proteins involved and the disease context. Due to variations in disease progression, genetic mutations or PTMs, the same protein may exhibit diverse structures through LLPS, including gel-like droplets, amorphous aggregates and amyloid fibrils. Ding et al. proposed a classification system for abnormal LLPS caused by disease mutations, categorizing them into two groups: loss of phase separation (LoPS) and gain of phase separation (GoPS) [46]. This classification helps to characterize condensates that exhibit limited or even disassembled phase separation capabilities, as well as newly acquired or transitional states of phase separation. Remarkably, the state of phase separation in disease is considerably more intricate, and this categorization is not absolute, as simultaneous losses and gains may occur (Fig. 2).

### 3.1 Tau LLPS drives the pathology of AD and other tauopathies

As a microtubule-associated protein, tau plays a vital role in the pathogenesis of a series of neurodegenerative diseases, including Alzheimer's disease (AD), progressive supranuclear palsy (PSP), corticobasal degeneration (CBD), Pick’s disease (PiD), and frontotemporal dementia with Parkinson's disease-17 (FTDP-17) [51]. Tau accumulates abnormally when hyperphosphorylated, decreasing the binding capacity of microtubules, making the cytoskeleton unstable and increasing the risk of various cognitive problems [52]. The ubiquitin-protease system is involved in the clearance of tau, with hyperphosphorylated tau binding to the proteasome to form neurofibrillary tangles (NETs), mostly in the cortical hippocampus and basal neurons. A strong correlation has been revealed between cognitive decline and the rate of tau aggregate formation. Interestingly, tau aggregates are seeded, spreading from one brain region to another, which suggests that this self-propagation is associated with a prion-like structure [53, 54].

There is growing evidence that tau executes several cellular functions by LLPS. In healthy neurons, tau can locally concentrate tubulin in the drops through phase separation and thus form microtubule bundles [55]. The transition from liquid to solid forms arises as a result of abnormal tau aggregation, and it is closely related to the pathological mechanisms of neurodegenerative diseases. Exactly how soluble tau protein transforms into aggregated tau is not fully understood. Tau separation from microtubules has been demonstrated to be a major initiator of tau pathology, and phase separation is thought to be the initial step in tau condensation [56]. Tau has no typical IDR in its protein sequence, and it exhibits phase separation in vitro in the presence of crowding agents due to the intrinsic disorder and uneven charge distribution in full-length tau [57]. Strictly speaking, tau is not subordinate to the RBP family, yet defects in RNA processing have been observed in patients suffering tauopathy [58]. It has been proposed that tau is closely related to RBPs with the ability to undergo phase separation. In the presence of RNA and/or protein chaperones, heterotypic tau LLPS (or complex coacervation) is driven by the attractive electrostatic interactions between them, and the RNA concentration determines whether droplets are formed because droplets dissolve at high RNA concentrations [59]. In AD and FTDP-17 patients or mouse models, RBPs such as TIA1 potentiate tau condensation [60-62].

Disease-associated mutations or abnormal PTMs at different sites form multivalent interactions and regulate tau LLPS towards an agglutinated state, subsequently leading to aggregation and sclerosis. Tau undergoes multiple PTMs, such as phosphorylation, acetylation, ubiquitination, glycosylation, SUMO-ization and methylation, among which phosphorylation plays pivotal roles in both physiology and pathology [56, 63, 64], and hyperphosphorylated tau is widely observed in AD patients [65]. In addition to damaging the assembly of microtubules, phosphorylated free tau continuously mislocalizes in dendrites and the cytosol, accumulating in response to pathological stress, promoting phase separation and ultimately leading to tau aggregation [66], which has been demonstrated in AD and FTDP-17 patients [67]. Pathogenic mutations have significant potential to regulate tau LLPS, which is usually manifested as a promoter effect. Interestingly, mutations in the MAPT (microtubule-associated protein tau) gene encoding tau on chromosome 17 induce FTDP-17 and promote the progression of the disease, which does not play a significant role in AD [63, 68, 69]. In vitro studies related to tau LLPS have shown that it can be facilitated by macromolecular crowding agents and high protein concentrations and that ionic strength and temperature also make a difference. Moreover, an imbalance in metal homeostasis promotes tau aggregation. In addition to increasing the toxicity of tau in cells directly [70], the dyshomeostasis of zinc ions (Zn2+) in the brain reduces the critical concentration of tau LLPS by increasing intermolecular interactions [71], accelerating tau condensation and ultimately leading to neuronal death [72, 73]. More examples have been proposed by Mukherjee S, who showed that Fe3+ significantly increases the formation of tau oligomers and protofibrils, which is closely related to the noncovalent interactions between the disordered structural domains of tau molecules that are enhanced and stabilized by Fe3+ [74]. Furthermore, tau coacervation can be driven by intermolecular electrostatic interactions between negatively charged polyelectrolytes of multivalent anionic cofactors such as RNA or heparin and positively charged middle and C-terminal parts [75].

**Figure 3.**
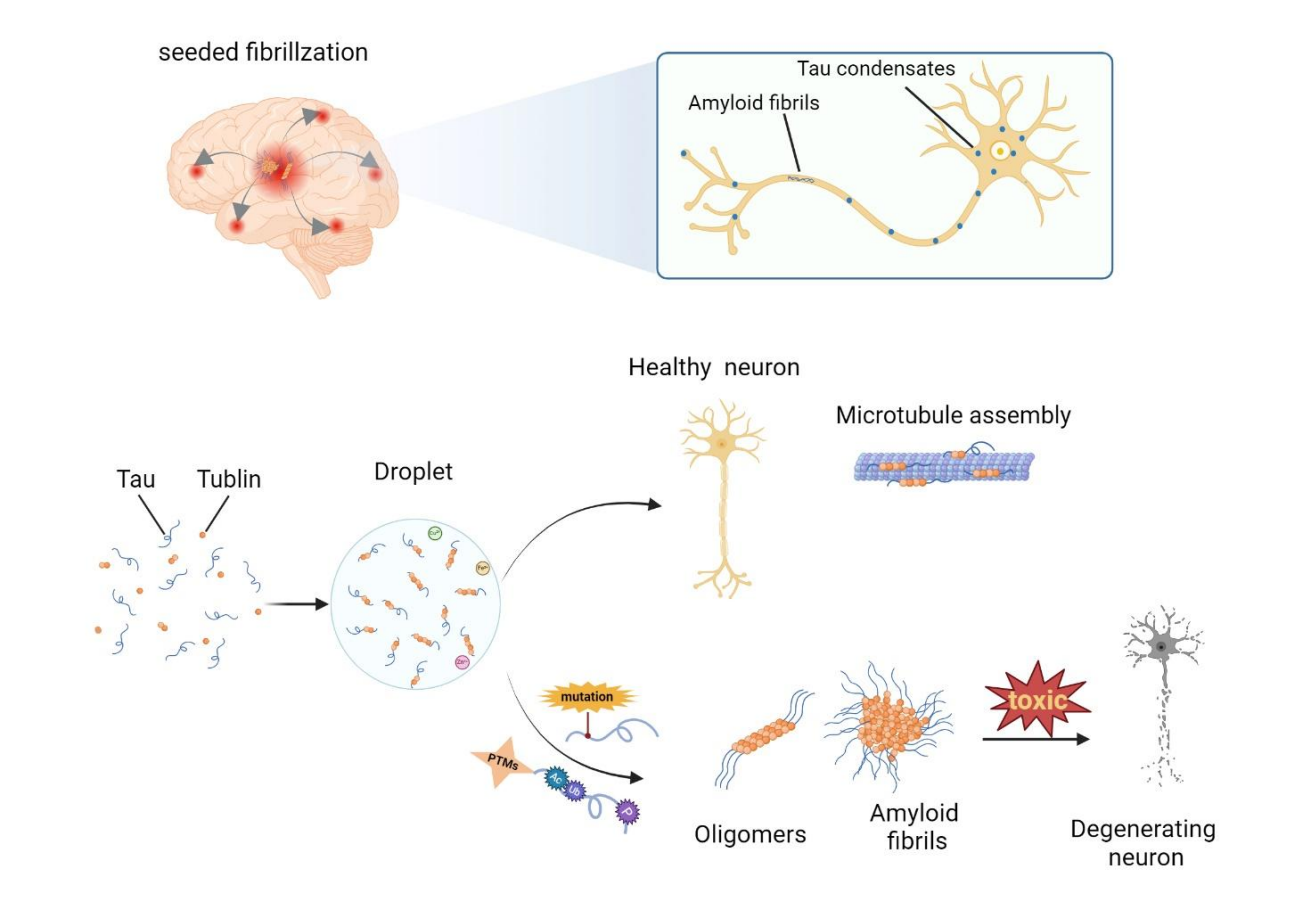
**Tauopathies driven by LLPS**. In healthy neurons, tau locally concentrates tubulin in the drops and assembles microtubule bundles. Abnormal PTMs (hyperphosphorylation, ubiquitination and acetylation) or disease-related mutations drive tau towards aggregation, and tau condenses self-propagates by a seeded polymerization mechanism.

In summary, studies on tau LLPS have grown vigorously. Tau droplets have been demonstrated to mature into pathological aggregates through aberrant LLPS and thus induce neuronal damage and inflammation. With more factors and regulators constantly being discovered and confirmed, a promising target for preventing tau aggregation is expected. The inhibitory effect of natural phenols on toxic tau aggregation has been demonstrated [76, 77], and gallic acid exhibits concentration-dependent biphasic modulation of tau LLPS [78]. Recently, researchers have successfully synthesized a small hybrid molecule by combining gallic acid and cyclic dipeptides, which effectively disrupts Fe3+-induced LLPS of tau and aids in attenuating abnormal tau fibrillation. This provides a new approach to interfere with tau aggregation [79] (Fig. 3).

### 3.2 RNA-binding protein LLPS: TDP-43 and FUS as paradigms in ALS and FTD

Amyotrophic lateral sclerosis (ALS) is a fatal neurodegenerative disease of unknown aetiology that primarily involves motor neurons in the cerebral cortex, brainstem and spinal cord. Frontotemporal dementia (FTD) is a disseminated or inherited form of dementia affecting the frontal and temporal lobes. It is the most common cause of neurodegenerative dementia other than Alzheimer's disease, including Pick's disease, and is a chronic, systemic and often irreversible decline in cognitive function.

TDP-43 and FUS are RNA-binding proteins (RBPs), and RNA is a key component involved in their phase separation. In addition to the IDRs of RNA-binding proteins [80], the standardized RNA-binding structural domains of RBPs also affect self-polymerization [81]. In addition, the dimerization domain harboured in RBPs mediates the formation of homologous or heterologous dimers, which further form oligomers that promote LLPS [82]. Alterations in subcellular localization affect the phase behaviour of proteins. RBPs such as TDP-43, FUS, and hnRNPA1/A2 are normally localized in the nucleus in a soluble single-state phase, where they bind with RNA and shuttle continuously between the nucleus and cytoplasm [47]. Unfortunately, when RBPs are mislocalized in the cytoplasm, insoluble solid pathological aggregates thus take shape and participate in the formation of stress granules (SGs), as several studies have suggested [83, 84]. These aggregates not only impair neuronal homeostasis and disrupt the regulation of nuclear RNA processing but also trigger several dysfunctions, such as mitochondrial dysfunction and inflammation. Accordingly, the nucleus is a reservoir containing high concentrations of RNA that make RBPs soluble, as proposed by Maharana, in which reduced RNA levels and abnormal changes in RNA-binding capacity lead to suspected phase transitions [85]. RNA mitigates excessive protein-protein interactions, which are responsible for the formation of pathological aggregates in various neurodegenerative diseases [86, 87]. In contrast, several studies have indicated that RNA binding mediates protein aggregation and neurotoxicity [88, 89]. Furthermore, aberrant LLPS caused by pathogenic mutations and overexpression of these proteins could interfere with the regulatory function of nuclear RNA processing, which, together with the toxicity of the aggregates themselves, ultimately leads to neurodegeneration.

TAR DNA-binding protein 43 (TARDBP 43, TDP-43) was the first protein identified to aggregate in ALS, the condensate of which can be found in the cytoplasm in almost all cases of ALS [90, 91]. Nuclear clearance and cytoplasmic aggregation of TDP-43 have been widely observed in damaged neurons and glial cells in both the spinal cord and brain lesions of ALS and tau-negative FTD patients. TDP-43 contains a nuclear localization signal (NLS) within its N-terminal domain (NTD) and a nonfunctional nuclear export signal (NES) in its RNA recognition motif (RMR) [47]. With a low-complexity C-terminal domain (CTD), TDP-43 exhibits a high propensity to aggregate during or immediately after purification. The sequestration of RNA-binding proteins induced by the accumulation of toxic RNAs contributes to disease aetiology [92, 93]. It has recently been reported that repeated CAG repeats result in elevated levels of N1-methyladenine (m1A), which binds to TDP-43 and subsequently facilitates the mislocalization of TDP-43 in the cytoplasm and the formation of gel-like aggregates [94]. The disease-causing mutations of TDP-43 are mostly located in CTDs [95, 96], implying that even minor changes in the IDR may make a large difference in neurodegeneration. These mutations impair the dynamics of membraneless organelles, leading to phase separation of disease-associated RNA-binding proteins, which in turn accelerates the progression of fibrosis and the formation of pathological amyloidogenic fibres deposited in the cell body and nerve plexus [97, 98].

Initially found to be related to liposarcoma, FUS is formed by chromosomal rearrangement followed by fusion with specific transcription factors. Later, the association with neurodegenerative diseases gradually appeared, with missense mutations in FUS being the cause of some ALS, and subsequently, FUS was identified in ubiquitinated protein inclusions in the brain tissue of some FTLD patients. FUS is a multifunctional RNA-binding protein primarily localized in the nucleus. FUS contains an NES within its RRM as well as an NLS within its CTD [48]. It is crucial to form dynamic liquid-like compartments through which FUS plays a physiological role [99, 100]. Studies have also noted that ALS-associated mutated FUS undergoes LLPS to form aggregates, which mislocalize in the cytoplasm [7]. In addition to pathogenic mutations, aberrant posttranslational modifications (PTMs) on RNA-binding proteins and their binding partners play important roles in the pathogenesis of ALS and FTD [101]. Studies have shown that the deletion of FUS arginine methylation promotes LLPS and reduces the dynamics of FUS cohesion in vitro and in cells based on the enhanced interaction between arginine and aromatic amino acid residues that drive FUS protein condensation, suggesting that PTMs can promote or inhibit the phase separation of disease-associated proteins [102, 103]. It is evident that targeting the respective PTM-modifying enzymes to affect key phase-separated proteins is a promising therapeutic strategy, as has been found for PAR polymerase inhibitors with neuroprotective effects in cellular and animal models of TDP-43-related toxicity [104].

**Figure 4.**
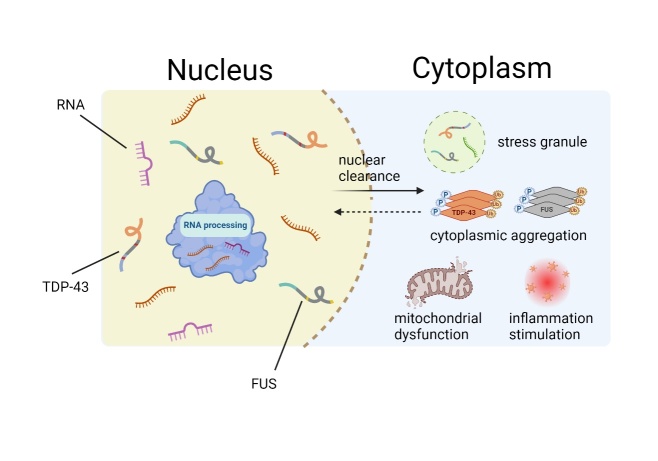
**Mislocalization and aggregation of TDP-43 and FUS LLPS**. TDP-43 and FUS are typically localized within the nucleus and play crucial roles in RNA processing. However, when mislocalized to the cytoplasm, they undergo aberrant liquid-liquid phase separation (LLPS), leading to the formation of cytoplasmic aggregates that trigger mitochondrial dysfunction and inflammatory stimulation.

Persistent stress granules can be observed in the spinal motor neurons of individuals with ALS. These granules, which are enriched with more than 200 proteins, are formed and bind to RNA in response to various cellular stressors, including oxidative stress and heat shock [105]. The release of RNA is expected upon the alleviation of stress, whereas the presence of misfolded proteins hinders this process. Under stressful conditions, proteins may undergo conformational changes that expose hydrophobic regions, thereby inducing the transition of stress granules from a liquid to a solid-like state [106]. Notably, although TDP-43 and FUS have been found to be recruited to SGs, previous research has also indicated that TDP-43 can form aggregates independently of SGs under cellular stress conditions [107, 108], suggesting that the precise role that they play in SGs remains elusive. The ubiquitin-protease system plays an important role in the clearance of SGs [40]. Additionally, molecular chaperones seem to be involved in the regulation of abnormal proteins. The molecular chaperone 70-kDa heat shock protein (HSP70) can prevent the accumulation of misfolded proteins in stress particles by chaperoning TDP-43 and FUS and can even breakdown stress particles be containing misfolded proteins [109, 110]. It has also been shown that the heat shock protein HSPB1 disassembles TDP-43 droplets and inhibits the assembly of TDP-43 into fibres, while a decrease in HSPB1 promotes the demixing and mislocalization of cytoplasmic TDP-43. HSPB1 deficiency containing aggregated TDP-43 was found in spinal cord motor neurons from ALS patients, confirming that HSPB1 is a regulator of cytoplasmic TDP-43 phase separation and aggregation [111].

As the most prominent examples of neurodegenerative diseases, ALS and FTD are not identical in terms of clinical manifestations, yet approximately half of FTD patients display TDP-43 pathology and share comparable molecular mechanisms with ALS patients, allowing us to regard them as having a spectrum of neurodegeneration with clinical and genetic similarities [99]. Multiple ALS pathogenic genes, such as TARDBP, FUS, and C9ORF72, have been identified as major pathogenic genes in FTD. Interestingly, abnormalities in the causative protein of familial FTD, tau, have been observed in the pathological tissues of ALS patients. Apart from tau protein, most of these pathogenic proteins are RBPs and are closely related to RNA stability. TDP-43, FUS, and C9ORF72 can undergo LLPS and form amyloid fibrils continuously during pathogenesis [112]. However, more in-depth research is required to understand more comprehensively how RBPs undergo nuclear clearance and mislocalize in the cytoplasm. Strategies to correct protein mislocalization and enhance compartment specificity could also be potential therapeutic approaches for treating neurodegenerative diseases such as ALS. Recent studies have demonstrated that different structural domains in TDP-43 could be exploited and targeted to develop drugs to stabilize the native state of TDP-43, which could constitute a viable and effective strategy for treating TDP-43 proteinopathies [113] (Fig. 4).

### 3.3 α-Syn LLPS: an alternative nucleation mechanism in PD

Parkinson's disease (PD) is a common motor disorder of the nervous system characterized by tremor, gait rigidity, posture instability and cognitive impairments. As the main pathological feature of PD, aberrant protein deposition causes the progressive loss of dopaminergic neurons, the main component of which is alpha-synuclein (α-Syn) inclusion bodies, also known as Lewy bodies (LBs) and Lewy neurites (LNs) [114, 115].

α-Syn is a soluble protein present in the presynaptic and perinuclear regions of the central nervous system. The binding of α-Syn to membranes allows it to display vital physiological functions in vesicle transport [116]. Generally, there is a dynamic balance between soluble monomeric α-Syn and its membrane-bound state [117]. Involved in the formation of the SNARE complex, α-Syn facilitates the fusion of synaptic vesicles and the presynaptic membrane to release neurotransmitters [118]. α-Syn is natively unstructured both in vitro and *in vivo*, whereas the α-helical conformation is observed when it is bound to lipid membranes [119]. The primary structure of α-Syn involves a central hydrophobic region named the nonamyloid β component (NAC), by which aggregation is mainly driven [50]. The N-terminal region possesses most of the familial mutations together with two LCDs, strongly supporting the idea that α-Syn LLPS occurs under appropriate conditions [120, 121]. Additionally, the C-terminal region, which is rich in proline, with most PTM sites located inside, binds ligands, small molecules, metal ions, etc., and contributes to the aberrant folding and aggregation of α-Syn [122]. α-Syn is often phosphorylated and ubiquitinated in pathological inclusions, where it forms higher-order amyloid fibrils with a cross-β-sheet-rich conformation to induce neurotoxicity [50, 123].

As a sporadic disease, PD is largely influenced by environmental factors such as metal ions, interactions with lipid membranes and cellular factor phosphorylation of Ser129 and familial mutations [124]. Previous research has shown that α-Syn senses lipid packing defects and has the highest affinity for lipids containing anions [125, 126]. With this affinity, α-Syn binds to membranes to perform a plethora of physiological functions, revealing the vital role of lipids and their interactions in α-Syn aggregation [127-129]. Binding with α-Syn stabilizes the liquid phase formed by negatively charged lipids, while the ratio of protein to lipid regulates α-Syn LLPS [130]. Although α-Syn can bind to different types of membranes with inherently soluble lipids, only lipids with the highest solubility in aqueous solution bind to α-Syn, leading to the formation of amyloid fibrils, which emphasizes the hypothesis that modifications of the chemical properties of lipids, such as those associated with oxidation and ageing, may play a crucial role in deciding whether the interactions between α-Syn and the membrane are beneficial or harmful. The presence of lipids with the shortest hydrocarbon chains enhances protein aggregation. For example, gangliosides, especially Gm1 and Gm3, have been demonstrated to accelerate the formation of α-Syn amyloids, with a similar situation being observed in AD. Notably, lipids are not indispensable for α-Syn LLPS and aggregation because aggregation occurs in the absence of lipids [131, 132].

α-Syn aggregation is also significantly influenced by metal ions. The pathogenesis of PD is closely related to iron metabolism [133]. Aggregation can be induced in iron-treated cells, probably because the accumulation of iron increases oxidative stress and further leads to lipid peroxidation [134, 135]. Previous studies by Soumik have demonstrated that α-Syn droplets are formed in cells under oxidative stress regulated by microtubules [136]. The strong binding of Fe2+ and Fe3+ transforms α-Syn into a β-sheet conformation and thus promotes aggregation, which is neurotoxic to neuronal cells [137]. Considering that calcium plays a role in the pathogenesis of PD and affects α-Syn assembly through interactions, Huang studied how Ca2+ regulates α-Syn aggregation and concluded that Ca2+ facilitates α-Syn phase separation to accelerate amyloid aggregation [138]. Owing to the increase in the multivalency of the polycation-protein interaction, other metal ions (Cu2+, Mn2+, Al3+, Co3+) have different effects on α-Syn LLPS and aggregation [139, 140]. The phosphomimetic substitution at serine 129 and the mutations A53T and A30P associated with PD pathogenesis have been shown to cause structural defects in membrane binding and to reduce the critical α-Syn concentration for LLPS, exhibiting an enhanced tendency to self-aggregate [141]. As mitochondrial impairment is common in PD patients, the amount of available ATP is largely limited, which affects α-Syn LLPS to some extent [142]. In addition, high concentrations of biogenic polyamines related to ageing have been shown to be involved in the pathogenesis of AD and PD and to affect protein aggregation to different degrees in neurons, with spermine (Sp) and spermidine (Spm) being typical examples related to the pathogenesis of PD [143, 144]. Researchers have also shown that α-Syn and full-length tau can interact for complex coacervation [145, 146].

The transition to aggregation and solidification of α-Syn cannot occur at one stroke, and studies indicate that α-Syn LLPS precedes its aggregation [136]. Subsequently, Poudyal proposed that oligomeric intermediates are indispensable before soluble monomeric α-syn eventually transforms into fibrillar aggregates of amyloids with a cross-β-sheet-rich conformation [147]. In recent years, α-Syn LLPS has been described as an alternative nucleation mechanism that acts as a reservoir to enrich concentrated α-Syn molecules, enhancing protein-molecule interactions and trapping toxic oligomeric intermediates and fibrils [136, 148].

### 3.4 Htt LLPS recruits other proteins and causes phase transition in HD

Huntington's disease (HD), also known as Huntington's chorea, is a debilitating autosomal dominant neurological condition for which effective disease-modifying treatments are lacking [149]. Huntington's disease is caused by expanded CAG triplet repeats at the N-terminus of the huntingtin (Htt) gene [149]. In the normal population, these trinucleotide repeats are also present, ranging from a few to approximately 50 repeats [150], and when the number of trinucleotide repeats exceeds a certain range, the Htt gene with a polyglutamine (polyQ) region can undergo erroneous splicing, the length of which is toxicity dependent [151, 152]. The N-terminal fragment (NTF) of the mutant Huntington's protein, the expanded polyglutamine (polyQ) region in the first exon, is directly responsible for the formation and deposition of the intracellular inclusion bodies found in the brains of affected patients [49]. It is unknown, however, how exactly the expansion of glutamine (CAG) repeats mediates the pathogenesis of HD.

Htt forms insoluble inclusions via LLPS, which affects cellular metabolism while also promoting the coaggregation and phase separation of other proteins from Htt-NTFs to weaken cells and cause apoptosis [153]. This finding suggested that the entire aggregation process of Htt-NTF aggregates has deleterious effects on neurons; that is, in addition to insoluble precipitation, soluble inclusions also cause neurotoxicity. Various posttranslational modifications of HTT, such as phosphorylation, acetylation and ubiquitination, are implicated in the pathogenesis of HD. Serine/threonine phosphorylation and lysine acetylation, for example, have been found to modulate HTT function and amplify HTT toxicity [154-157]. Arginine methylation has been shown to affect proteolysis, phase transition and aggregation. Tamara's finding that altered arginine methylation sites in HTT greatly reduce its solubility in cells suggests a potential effect of HTT methylation on its aggregation pathway, and it is hypothesized that methylation of the R586/50 site plays a major role in the HTT phase transition [158].

Htt has a large relative molecular weight (~350 kDa) and can act as a scaffold to recruit many other proteins to interact [159]. These 'clients', or 'ligands', can change saturation concentrations and move phase boundaries to stabilize or disrupt specific phases [160]. In this case, Posey et al. found that an abundant cellular protein, Profilin, binds directly to the C-terminal Pro-rich region of Htt-NTF (and preferentially binds monomers or oligomers of Htt-NTF) and moves the phase separation concentration boundary to a higher value through a process known as polyphasic linkage, thereby destabilizing aggregation and phase separation and ultimately reducing Htt-NTF aggregation and toxicity in the cell [161].

Considering the polyphasic linkage in HTT LLPS and the cytotoxicity of more than one type of aggregate species and the presence of unknown ligands with preferential binding, more work should be done to provide an adequate picture of the overall aggregation process.

## 4. Concluding remarks and future perspectives

The manifestations of neurodegenerative diseases are diverse, and most of them are not effectively treated. Interestingly, the representative protein aggregates of various neurodegenerative diseases may share some common components and functions to a certain extent. More than half of autopsies in elderly individuals coexist with multiple neurodegenerative diseases of different aetiologies, and tau and α-Syn aggregates cooccur in LB variants of AD, DLB (dementia with Lewy bodies) and PD with dementia [162], implying the importance of targeting aggregates for treatment. Considering that LLPS droplets are a major intermediate in protein aggregation, disruption of this process represents a very promising target for developing novel therapeutic options. By altering the localization of specific condensate community members or altering physiochemical properties, condensates can be dissolved or induced, which provides us with more opportunities to intervene and treat diseases by focusing on the typical disease-related phase separation process.

For traditional pharmaceuticals, drug design and characterization are largely limited by the structure of potential protein targets [163]. As Avinash proposed, the emergence of condensate modifiers (c-mods), such as small molecule inhibitors, antibodies and synthetic peptides, will enable the treatment of traditional undruggable proteins [164]. It has been indicated that condensates can be modulated by being dissolved or induced, altering the localization of specific condensate community members or altering physiochemical properties without the need for specific binding pockets as targets [165], and a wide range of diseases sharing a common pathology will benefit from these new therapies. Compounds with planar moieties such as mitoxantrone and pyrvinium have been demonstrated to dissolve persistent stress granules in cells by binding to nucleic acids and potentially to IDRs, preventing the progression of ALS [166]. The small molecule ISRIB (and its analogues) can reverse phosphorylated eIF2α-dependent SG formation and modulate interactions within the condensate community [167, 168]. Additionally, conventional treatments primarily target the aggregated protein form to inhibit its formation or counteract its pathogenic characteristics. For neurodegenerative diseases, however, it is more favourable to maintain the stability of disease-specific proteins in their native state than to eliminate aggregates. This approach not only mitigates the toxic effects gained by protein aggregation but also addresses potential detrimental consequences arising from loss of normal protein function. Recently, Yang indicated that the modulation of some specific sites (S333D/S342D) or binding to small-molecule stabilizers stabilizes monomeric TDP-43 without altering its physiological properties, which represents a step further in disease treatment [113]. C-mods provide us with a new perspective for targeting undruggable proteins and addressing a wide array of genotypes with the same disease phenotype using a single therapeutic strategy. However, on the road to fully displaying the advantages of c-mods, several challenges and questions remain. An overall understanding of the composition of condensates is crucial, and developing and identifying c-mods in an easy yet accurate manner remains a challenging task. The specificity of c-mods is also not guaranteed, and it is difficult to determine whether small molecules designed for a specific condensate affect other unrelated condensates. Furthermore, more efforts should be made to construct widely accepted and effective drug screening and development platforms designed to address abnormal phase separation in biological systems.

Further studies on aberrant condensation and the transition to pathological fibrillar aggregates will enhance our understanding of the molecular principles underlying the initiation and progression of neurodegenerative diseases. The mechanisms underlying the toxicity and fibrosis of neurodegenerative disease-specific proteins have been extensively and thoroughly investigated; however, there is still ongoing controversy regarding the identification and determination of whether these proteins undergo LLPS. Biomolecular condensates are subcellular structures with lengths ranging from nanometres to micrometres, and microscopy methods for observing and identifying them are needed. By observing whether a protein expressed in cells or reconstituted and purified in vitro can form spherical droplets as well as the mobility of the droplets, researchers have assessed its potential for phase separation [9]. Advances in electron and fluorescence microscopy have led to the emergence of membrane-less assemblies as another paradigm for cellular compartmentalization, with more MLOS being reported in recent decades. While the liquid-like nature of MLOS in live cells can be observed using time-lapse light microscopy imaging and many researchers tend to develop a simple system in vitro to study the properties of proteins with the ability to condense, in most cases, minor changes in the microenvironment can trigger a sharp, instantaneous response, leading to phase separation being difficult to capture by direct observation. Apart from microscopy, which focuses on the morphological characterization of MLOS, other experimental methods, such as FRAP, have been applied to explore the rheology of biomolecular condensates. Researchers have also attempted to use high-throughput, mass spectrometry-based methods to perform comprehensive analyses of protein-protein interaction networks [169, 170] to identify the nucleolar proteome [171]. Other methods, such as NMR spectroscopy, solid-state nuclear magnetic resonance spectroscopy, X-ray diffraction, and scattering methods, have been developed to further study the structure, dynamics, and biology of membrane-less organelles. Given the infancy of the field, more efforts should be made to develop technical means to characterize the thermodynamic, kinetic, and structural properties of proteins and promote studies on LLPS. Considering the technological limitations of spatial and temporal resolution as well as biological complexity, more complementary approaches and characterization methods must be developed and integrated to address questions related to the structure, dynamics, and biology of MLOS.

Through research on LLPS in diseases in recent years, we can easily find that most researchers only focus on famous disease-specific proteins or proteins with a typical IDR. In addition to the aforementioned proteins, further investigations of biomolecules exhibiting phase separation potential, as well as improved identification of partners in highly dynamic contexts, are warranted. Despite the flourishing research on finding new evidence of LLPS, there is no acknowledged conclusion on the discrimination of condensates. The pressing questions at hand include the verification of observed structures as condensates within living cells and the distinction between different intracellular condensates. Functional research on LLPS in diseases is still in the early stages because most of the existing research is limited to identifying this phenomenon. The elucidation of its molecular mechanism has posed a formidable challenge due to the intricate interplay between its macroscopic properties and microstructure. Additional studies are required to more comprehensively understand the key tenets of the precise role that LLPS plays in the mechanism of diseases. Moreover, there is a data gap pertaining to the clinical application of LLPS, and the association of the theory of LLPS with clinical results is a worthwhile endeavor that merits our efforts. There is a promise for more breakthroughs in incurable diseases, as the internal mechanism of LLPS and diseases are excavated in the future.
